# Inclisiran in advanced chronic kidney disease (stage G4–G5 non-dialysis): sustained LDL-C reduction and favorable safety profile at 1-year follow-up in a real-world case series

**DOI:** 10.3389/fmed.2026.1881641

**Published:** 2026-06-22

**Authors:** Avinash Chandu Nanwani, Jose Carlos De la Flor Merino, Elena Jiménez Mayor, Juan Daniel Díaz García, Celia Rodríguez Tudero, Arturo Villalobos Navarro, Esperanza Moral Berrio, Adriana Puente García, Jesus Hernández Vaquero

**Affiliations:** 1 Hospital General de Fuerteventura, Fuerteventura, Canarias, Spain; 2 Hospital Central de la Defensa Gomez Ulla, Madrid, Spain; 3 Hospital Santa Barbara, Soria, Spain; 4 Hospital General de Mexico Dr. Eduardo Liceaga, Mexico City, Mexico; 5 Hospital Universitario de Salamanca, Salamanca, Spain; 6 Hospital Barros Luco Trudeau, San Miguel, Chile; 7 Hospital General Universitario de Ciudad Real, Ciudad Real, Spain; 8 Hospital Universitario de Fuenlabrada, Fuenlabrada, Spain

**Keywords:** advanced chronic kidney disease, albuminuria, cardiovascular risk, inclisiran, LDL-cholesterol, Lipoprotein(a)

## Abstract

**Background and Aims:**

Advanced chronic kidney disease (CKD; stages G4–G5 not on dialysis) is associated with very high cardiovascular risk, and lipid control is particularly challenging due to reduced statin efficacy and frequent intolerance. Inclisiran, a small interfering RNA (siRNA) that inhibits hepatic PCSK9 synthesis, has demonstrated sustained LDL-C reductions in clinical trials, though data in advanced CKD remain scarce.

**Materials and methods:**

An observational, retrospective case series was conducted at a tertiary care hospital, including 10 patients with stage G4–G5 non-dialysis CKD and elevated LDL-C despite optimized therapy or with statin intolerance. Inclisiran was administered following the standard regimen (day 0, day 90, and every 6 months thereafter). Lipid profile, renal function, and albuminuria were assessed at baseline, 3–6 months, and 12 months.

**Results:**

Patients had a mean age of 75 ± 5 years with high cardiovascular comorbidity. Baseline LDL-C was 131.6 ± 22.1 mg/dl, falling to 46.1 ± 26.5 mg/dl at 1 year (mean reduction 65.6%). All patients responded to treatment; 80% achieved LDL-C < 70 mg/dl and 60% achieved < 55 mg/dl. Renal function remained stable (eGFR 22.7 vs. 23.7 ml/min/1.73 m^2^). A 27.4% reduction in median albuminuria was observed; however, all patients were receiving maximized nephroprotective therapy concurrently, and this finding should not be attributed to inclisiran. No adverse events or major cardiovascular events were recorded.

**Conclusions:**

Inclisiran demonstrates a marked and sustained LDL-C reduction in patients with advanced CKD, with an excellent safety profile and preserved renal function, supporting its use in this high-risk population.

## 1 Introduction

Advanced chronic kidney disease (CKD) is associated with a markedly increased cardiovascular risk, driven by the interplay of chronic inflammation, endothelial dysfunction, oxidative stress, lipid metabolism abnormalities, and the accumulation of uremic toxins (1, 2). These pathophysiological mechanisms accelerate atherosclerosis and substantially increase the incidence of major adverse cardiovascular events (MACE), particularly in patients with CKD stages 4 and 5 (3).

Despite often normal LDL-C levels in advanced CKD, LDL particles become more atherogenic due to a shift toward smaller, denser, and oxidation-prone forms with enhanced arterial wall penetration. CKD is also characterized by impaired clearance of triglyceride-rich apoB-containing lipoproteins, leading to accumulation of VLDL and remnant particles. At the same time, HDL-C levels decline and HDL functionality deteriorates, diminishing cholesterol efflux and anti-inflammatory activity and further promoting plaque formation (3).

Current KDIGO and ESC/EAS guidelines recommend statin therapy, with or without ezetimibe, to reduce cardiovascular risk in patients with non-dialysis CKD (4, 5). However, the effectiveness of conventional lipid-lowering therapy may be limited in advanced CKD. Statin intolerance is relatively common in this population, and achieving guideline-recommended LDL-C targets (< 55 mg/dl or ≥50% reduction from baseline) is often challenging, underscoring the difficulty of attaining adequate lipid control with conventional therapies.

Inclisiran, a small interfering RNA (siRNA) targeting hepatic synthesis of proprotein convertase subtilisin/kexin type 9 (PCSK9), has demonstrated sustained LDL-C reductions of approximately 50% in phase III clinical trials, including ORION-9, ORION-10, and ORION-11 (6–8). Long-term extension studies such as ORION-3 and ORION-8 have confirmed the durability of this lipid-lowering effect and the favorable safety profile of the drug over extended follow-up periods (9). In addition, the twice-yearly dosing schedule may improve treatment adherence, an important consideration in patients with advanced CKD who frequently experience a high therapeutic burden and polypharmacy.

Despite these promising findings, evidence specifically addressing patients with severe renal impairment remains limited. Patients with an estimated glomerular filtration rate (eGFR) below 30 ml/min/1.73 m^2^ were either underrepresented or excluded from pivotal trials, leaving uncertainty regarding the efficacy, renal safety, and clinical utility of inclisiran in this high-risk population (6–9). Pharmacokinetic studies suggest that renal dysfunction has only a modest effect on inclisiran exposure (10); however, real-world data are needed to confirm its effectiveness and safety in patients with advanced CKD, particularly with respect to kidney function and albuminuria.

The aim of the present study was to describe real-world experience with inclisiran in a cohort of patients with stage G4–G5 CKD not receiving dialysis, focusing on lipid-lowering efficacy, renal outcomes, and treatment safety during follow-up.

## 2 Materials and methods

### 2.1 Study design

This retrospective observational study was designed as a real-world case series conducted at a tertiary care hospital. Data were extracted from electronic medical records in accordance with the ethical principles of the Declaration of Helsinki (11).

### 2.2 Study population

The study included 10 consecutive adult patients (≥18 years) with advanced CKD (stage G4 or G5 not receiving dialysis), defined according to KDIGO criteria (4) as an estimated glomerular filtration rate (eGFR) below 30 ml/min/1.73 m^2^ calculated using the CKD-EPI equation (12). All patients initiated treatment with inclisiran during the study period in the nephrology outpatient clinic of Hospital Gómez Ulla (Madrid). Clinical and laboratory data were collected at baseline, the first visit between 3 and 6 months (V1) after initiation of inclisiran treatment, and the second visit at 12 months after treatment initiation (V2).

### 2.3 Inclusion criteria

Patients were eligible if they were aged ≥18 years and had stage 4–5 CKD not requiring dialysis, as defined by the CKD-EPI equation. Additional inclusion criteria included a persistent elevation of LDL-C despite optimized lipid-lowering therapy–defined as treatment with a maximally tolerated statin dose, ezetimibe, bempedoic acid, and/or PCSK9 inhibitors—as well as documented statin intolerance, in accordance with KDIGO and ESC/EAS recommendations (4, 5). The availability of baseline laboratory data and at least one follow-up measurement between 3 and 12 months after the first inclisiran dose was also required. Patients were included regardless of the presence or absence of proteinuria.

Patients with CKD stages 3a−3b, history of kidney transplantation, concurrent acute kidney injury or follow up shorter than 6 months were excluded.

### 2.4 Intervention

Inclisiran was administered at a dose of 284 mg subcutaneously, according to the approved dosing schedule used in the ORION trials (6–8): an initial dose on day 0, a second dose on day 90, and subsequent doses every 6 months. Concomitant lipid-lowering therapies, including statins, ezetimibe, bempedoic acid, and PCSK9 inhibitors, were recorded, as well as the presence of statin intolerance.

### 2.5 Data collection

#### 2.5.1 Clinical and demographic variables

Baseline variables included age, sex, comorbidities (hypertension, diabetes mellitus, ischemic heart disease, prior stroke or transient ischemic attack), smoking status, obesity, and CKD etiology. Obesity was classified according to body mass index (BMI) following World Health Organization criteria as BMI > 30.0 kg/m^2^.

#### 2.5.2 Kidney function

Serum creatinine and eGFR (CKD-EPI) were recorded at baseline, 3–6 months, and 12 months. Albuminuria was assessed using the urine albumin-to-creatinine ratio (ACR) when available. Changes in kidney function were classified according to validated criteria (13) as improvement (≥10% increase in eGFR), stability (±10% variation), or deterioration (≥10% decrease).

#### 2.5.3 Lipid profile

LDL-C, HDL-C, triglycerides, total cholesterol, apolipoprotein B100 (ApoB100), and lipoprotein(a) were recorded at the same time points. Lipid targets were defined according to ESC/EAS guidelines (5) as LDL-C ≤ 55 mg/dl or a ≥50% reduction from baseline.

#### 2.5.4 Safety assessment

Adverse events, hospitalizations, major cardiovascular events, and initiation of kidney replacement therapy were recorded. Adverse events were classified according to criteria used in the ORION trials (6–8).

### 2.6 Statistical analysis

Given the small sample size, only descriptive statistics were performed. Continuous variables are presented as mean ± standard deviation or median (interquartile range), whereas categorical variables are expressed as frequencies and percentages.

Primary outcomes included absolute and relative changes in LDL-C levels, the proportion of patients achieving ≥50% LDL-C reduction, and the proportion achieving LDL-C levels below 55 mg/dl.

Secondary outcomes included changes in eGFR, variation in albuminuria, treatment safety and tolerability, as well as the evolution of the remaining lipid profile parameters [HDL-C, total cholesterol, triglycerides, apolipoprotein B100, and lipoprotein(a)].

No inferential statistical analyses were conducted, in line with methodological recommendations for case series studies (14).

## 3 Results

Ten patients with advanced CKD (stages G4–G5 not receiving dialysis) were included. The mean age was 75 ± 5 years, and 70% were male. The cohort had a high burden of cardiovascular comorbidity: all patients had hypertension and ischemic heart disease, while 90% had diabetes mellitus and 50% had a history of stroke or transient ischemic attack. Additionally, 80% were obese and 60% were current or former smokers.

Five patients (50%) had documented statin intolerance, and most were receiving concomitant ezetimibe therapy. Baseline eGFR was 22.7 ± 6 ml/min/1.73 m^2^ and baseline LDL-C was 132 ± 22 mg/dl. Additional baseline clinical and laboratory characteristics are summarized in Table 1.

**Table 1:** Baseline clinical and laboratory characteristics of the study cohort.

Baseline characteristics	Total (*n* = 10)
Age (years), mean ±*SD*	75 ± 5
Male sex, *n* (%)	7 (70%)
CKD stage (non-dialysis), *n* (%)	Stage G4: 8 (80%); stage G5 ND: 2 (20%)
CKD etiology—diabetes mellitus, *n* (%)	6 (60%)
CKD etiology—vascular/NAE, *n* (%)	2 (20%)
CKD etiology—tubulointerstitial nephritis, *n* (%)	2 (20%)
Hypertension, *n* (%)	10 (100%)
Diabetes mellitus, *n* (%)	9 (90%)
Ischemic heart disease, *n* (%)	10 (100%)
Prior stroke/TIA, *n* (%)	5 (50%)
Peripheral artery disease, *n* (%)	1 (10%)
Obesity (BMI > 30 kg/m^2^), *n* (%)	8 (80%)
Smoking (current or former), *n* (%)	6 (60%)
Statins intolerance, *n* (%)	5 (50%)
Ezetimibe therapy, *n* (%)	9 (90%)
Bempedoic acid therapy, *n* (%)	2 (20%)
Prior PCSK9 inhibitor therapy, *n* (%)	1 (10%)
Baseline total cholesterol mg/dl, mean ±*SD*	198.9 ± 30.2
Baseline HDL cholesterol mg/dl, mean ±*SD*	49.9 ± 10.1
Baseline non-HDL cholesterol mg/dl, mean ±*SD*	149.0 ± 26.6
Baseline LDL-C (mg/dl) mean ±*SD*	132 ± 22
Baseline triglycerides mg/dl, mean ±*SD*	147.3 ± 63.4
Baseline ApoB100 mg/dl, mean ±*SD*	112.3 ± 15.2
Baseline Lp(a) mg/dl, mean ±*SD*	57.7 ± 24.4
Baseline HbA1c %, mean ±*SD*	6.6 ± 1.0
Baseline creatinine mg/dl, median (IQR)	2.3 [2.2–2.8]
Baseline eGFR ml/min/1.73 m^2^, mean ±*SD*	22.7 ± 6
Baseline albuminuria (ACR, mg/g), median (IQR)	220 [110–350]

SD, standard deviation; CKD, chronic kidney disease; ND, non-dialysis; NAE, nephroangiosclerosis; TIA, transient ischemic attack; BMI, body mass index; PCSK9, proprotein convertase subtilisin/kexin type 9; HDL, high-density lipoprotein; non-HDL, non-high-density lipoprotein; LDL-C, low-density lipoprotein cholesterol; ApoB100, apolipoprotein B100; Lp(a), lipoprotein(a); HbA1c, glycated hemoglobin; eGFR, estimated glomerular filtration rate; ACR, albumin-to-creatinine ratio; IQR, interquartile range.

Regarding lipid-lowering efficacy, LDL-C decreased from 131.6 ± 22.1 mg/dl at baseline to 51.4 ± 25.5 mg/dl at the first follow-up visit (V1) and 46.1 ± 26.5 mg/dl at the second follow-up visit (V2). The mean absolute change at V2 was −85.5 mg/dl, corresponding to a mean relative reduction of 65.6%. At the final follow-up, all patients (10/10) achieved a ≥30% reduction in LDL-C, and six patients (60%) achieved a ≥50% reduction. In addition, eight patients (80%) reached LDL-C levels < 70 mg/dl, and six patients (60%) achieved levels < 55 mg/dl. ApoB100 also decreased substantially, from 112.3 ± 15.2 mg/dl at baseline to 57.3 ± 20.0 mg/dl at 6 months and 53.6 ± 21.0 mg/dl at 12 months, consistent with a reduction in atherogenic lipoprotein burden. Triglycerides showed a moderate reduction, from 147.3 ± 63.4 mg/dl at baseline to 111.6 ± 37.1 mg/dl at 6 months and 111.1 ± 35.1 mg/dl at 12 months. HDL cholesterol remained relatively stable over time, with values of 49.9 ± 10.1 mg/dl at baseline, 48.5 ± 13.6 mg/dl at 6 months, and 45.7 ± 12.3 mg/dl at 12 months. Lp(a) showed a moderate numerical decrease, from 57.7 ± 24.4 mg/dl at baseline to 49.0 ± 20.8 mg/dl at 6 months and 46.6 ± 19.7 at 12 months, corresponding to mean relative reductions of approximately 15.1% and 19.2%, respectively. Lp(a) was measured using an isoform-insensitive immunoturbidimetric assay and reported in mg/dl.

Overall, inclisiran was associated with a marked and sustained reduction in LDL-C and ApoB100 over 12 months, with parallel reductions in total cholesterol, triglycerides, and Lp(a), while HDL cholesterol remained broadly stable.

**Figure 1:**
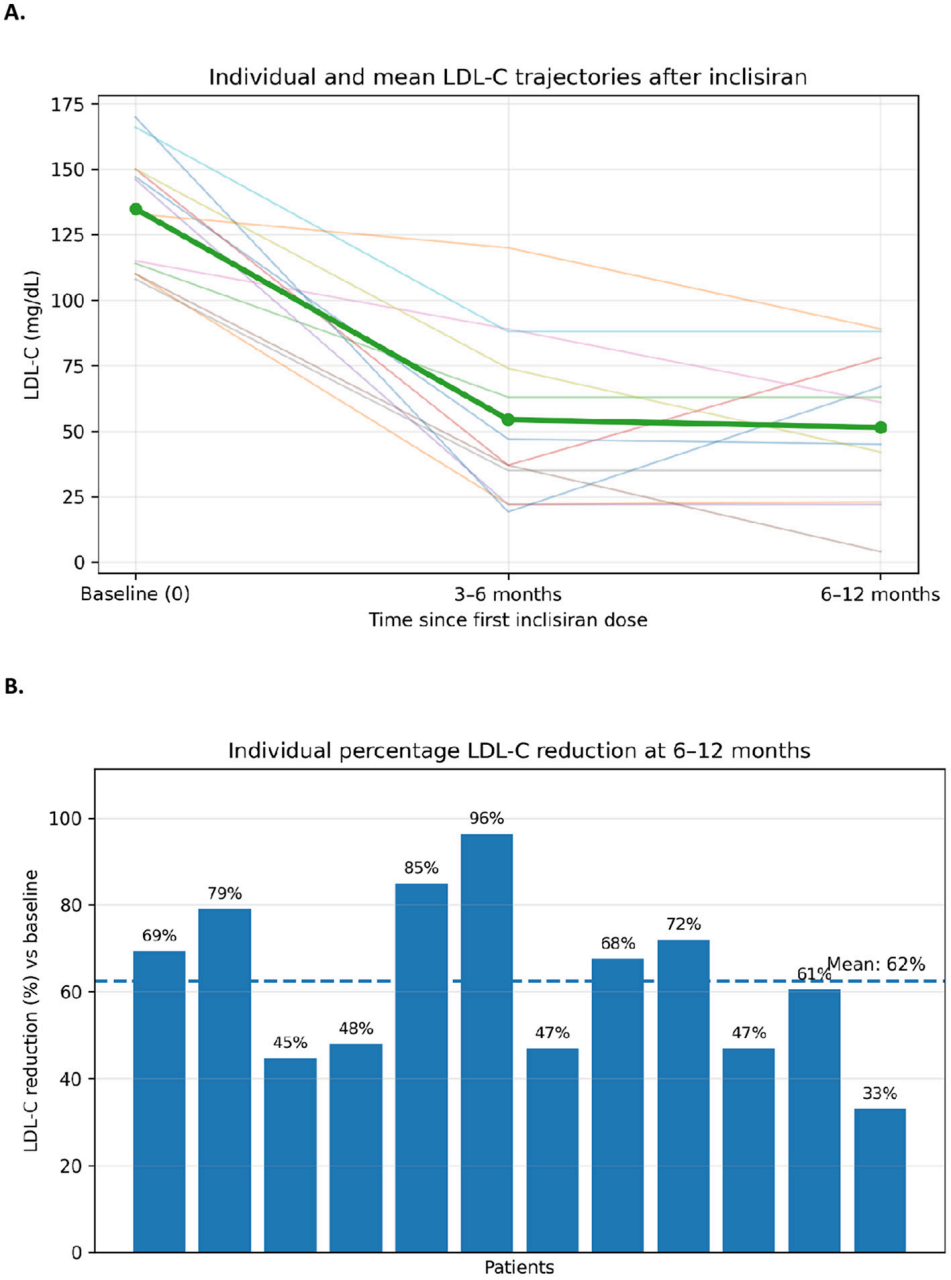
**(A)** Individual and mean LDL-C trajectories after inclisiran. Changes in LDL-C levels from baseline to 3–6 months and 6–12 months after the first inclisiran dose. Thin lines represent individual patients, and the thick line represents the group mean. **(B)** Individual percentage reduction in LDL-C at visit 2 (V2) compared with baseline.

The evolution of the lipid profile during follow-up is presented in Table 2, and the individual percentage reduction in LDL-C is illustrated in Figures 1A,B.

**Table 2:** Evolution of lipid profile and kidney function.

Parameter	Baseline	V1 (3–6 months)	V2 (12 months)
Total cholesterol mg/dl, mean ±*SD*	198.9 ± 30.2	116.7 ± 30.6	110.5 ± 34.6
LDL cholesterol mg/dl, mean ±*SD*	131.6 ± 22.1	51.4 ± 25.5	46.1 ± 26.5
HDL cholesterol mg/dl, mean ±*SD*	49.9 ± 10.1	48.5 ± 13.6	45.7 ± 12.3
Triglycerides mg/dl, mean ±*SD*	147.3 ± 63.4	111.6 ± 37.1	111.1 ± 35.1
ApoB100, mg/dl, mean ±*SD*	112.3 ± 15.2	57.3 ± 20.0	53.6 ± 21.0
Lp(a) mg/dl, mean ±*SD*	57.7 ± 24.4	49.0 ± 20.8	46.6 ± 19.7
Creatinine (mg/dl)	2.56 ± 0.56	2.58 ± 0.62	2.53 ± 0.65
eGFR (ml/min/1.73 m^2^)	22.7 ± 5.5	22.6 ± 6.2	23.7 ± 7.5
Albuminuria ACR (mg/g), median (IQR)	220 (110–350)	170 (98–345)	128 (91–272)

V1, first follow-up visit (3–6 months); V2, second follow-up visit (12 months); SD, standard deviation; LDL-C, low-density lipoprotein cholesterol; HDL-C, high-density lipoprotein cholesterol; ApoB100, apolipoprotein B100; Lp(a), lipoprotein(a), eGFR, estimated glomerular filtration rate; ACR, albumin-to-creatinine ratio; IQR, interquartile range.

Regarding renal outcomes, kidney function remained globally stable throughout the follow-up period. Mean estimated glomerular filtration rate (eGFR) changed from 22.7 ± 5.5 ml/min/1.73 m^2^ at baseline to 23.7 ± 7.5 ml/min/1.73 m^2^ at V2. According to the predefined criteria for relative change in eGFR (±10%), 20% of patients showed improvement, 70% remained stable, and 10% experienced deterioration.

**Figure 2:**
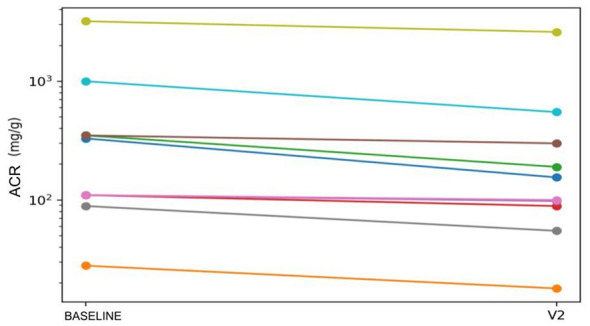
Individual evolution of albuminuria (ACR) between baseline and V2 (logarithmic scale).

Albuminuria showed a decreasing trend during follow-up. Median urine albumin-to-creatinine ratio (ACR) declined from 220 mg/g (IQR 110–350) at baseline to 128 mg/g (IQR 91–272) at V2, corresponding to a median relative reduction of −27.4%. Notably, this improvement occurred in patients who were already receiving maximally tolerated renin–angiotensin system blockade and SGLT2 inhibitors at study inclusion, with some additionally treated with finerenone, indicating that the reduction in albuminuria took place despite optimized background nephroprotective therapy. These results are presented in Table 2, while the individual trajectories of albuminuria are shown in Figure 2.

Regarding safety, no local or systemic adverse events attributable to inclisiran were observed during the follow-up period. No treatment discontinuations occurred, and no major cardiovascular events or renal events were recorded. Overall, inclisiran demonstrated a favorable safety and tolerability profile in this cohort of patients with advanced CKD (G4–G5 not receiving dialysis).

## 4 Discussion

Patients with advanced CKD represent a population at extremely high cardiovascular risk, with cardiovascular disease remaining the leading cause of morbidity and mortality in this group (1, 2). This excess risk reflects the complex interaction between traditional cardiovascular risk factors and CKD-specific mechanisms, including chronic inflammation, oxidative stress, endothelial dysfunction, vascular calcification, and metabolic disturbances (1, 3).

Despite this substantial cardiovascular burden, patients with advanced CKD have historically been underrepresented or excluded from major clinical trials evaluating lipid-lowering therapies (4, 5). Consequently, evidence guiding optimal lipid management in patients with eGFR < 30 ml/min/1.73 m^2^ remains limited, and therapeutic goals are frequently difficult to achieve in routine clinical practice (6).

In this real-world case series including exclusively patients with advanced CKD (stages G4–G5 not on dialysis), inclisiran produced a substantial and sustained reduction in LDL-C levels, comparable to the magnitude reported in pivotal phase III ORION trials (8, 15). Importantly, this lipid-lowering effect was observed in a cohort characterized by high cardiovascular comorbidity and frequent statin intolerance, clinical scenarios commonly encountered in advanced CKD and often associated with limited therapeutic options (4, 10).

In our advanced non-dialysis CKD cohort, inclisiran was also associated with reductions in lipoprotein(a) [Lp(a)] and apolipoprotein B-100 (apoB-100), suggesting a broader lowering of atherogenic lipoprotein burden beyond LDL-C. This observation is consistent with a *post hoc* pooled analysis of the phase III ORION-9/10/11 trials, in which inclisiran produced significant decreases in apoB and Lp(a) across all baseline eGFR strata, including patients with eGFR as low as 15–45 ml/min/1.73 m^2^, supporting the preservation of these effects even in more advanced CKD stages.

In this context, it is worth mentioning that the dual reduction of LDL-C and Lp(a) may be associated with a marked improvement in the mechanical vascular profile and is best interpreted when lipid changes are linked to instrumental markers of vascular function. For example, a recent real-world study demonstrated that the combined reduction of LDL-C and Lp(a) with PCSK9 inhibitors was associated with an improvement in pulse wave velocity (16), supporting the idea that the benefit of lipid reduction may extend beyond achieving the biochemical target and may involve measurable vascular effects. Such vascular assessment was not performed in our study; however, this could be discussed as a potential avenue of research for future studies in advanced chronic kidney disease.

Mechanistically, apoB-100 provides a direct measure of circulating atherogenic particle number (including LDL and the LDL-like Lp(a) particle), and its reduction may be particularly informative in CKD, where discordance between LDL-C (cholesterol mass) and particle burden can occur. Contemporary meta-analytic evidence further supports a modest but consistent Lp(a)-lowering effect with inclisiran (approximately 18% on average), alongside reductions in LDL-C and apoB, although the incremental contribution of Lp(a) lowering to clinical outcomes remains uncertain. It should be noted that the Lp(a) reduction observed in our cohort is a descriptive finding in a small sample and should not be interpreted as a proven clinical benefit. Rather, it should be framed within the broader context of residual lipid-related cardiovascular risk: even moderate reductions in Lp(a) may be relevant as part of a comprehensive atherogenic lipoprotein-lowering strategy in patients with extreme cardiovascular risk, particularly while awaiting dedicated Lp(a)-targeted therapies. The clinical significance of this finding warrants confirmation in larger, prospective studies (17).

A particularly relevant aspect of our study is that all included patients had an eGFR below 30 ml/min/1.73 m^2^. This population was largely excluded from pivotal phase III trials evaluating inclisiran. In both ORION-10 and ORION-11, patients with severe renal impairment and eGFR < 30 ml/min/1.73 m^2^ were excluded, resulting in very limited safety and efficacy data for inclisiran in advanced CKD (8). This lack of evidence was one of the main motivations for conducting and reporting the present study, aiming to provide real-world data regarding the safety and tolerability of inclisiran in this particularly vulnerable population.

From a renal safety perspective, kidney function remained globally stable during follow-up, with no major renal adverse events observed. The small proportion of patients who experienced deterioration in kidney function likely reflects the expected natural progression of advanced CKD rather than a direct drug-related effect. These findings are consistent with previous pharmacokinetic analyses suggesting that renal impairment has minimal impact on inclisiran exposure, efficacy, or tolerability (11).

Our observations are also consistent with emerging real-world evidence evaluating inclisiran in CKD populations. A recently published Italian real-world study evaluating inclisiran across different stages of CKD reported substantial LDL-C reductions, stable renal function, and no major adverse events during follow-up (18). However, only five patients included in that cohort had an eGFR below 30 ml/min/1.73 m^2^, highlighting the persistent scarcity of data specifically focused on patients with advanced CKD. In this context, our study contributes additional clinically relevant real-world evidence in a cohort composed exclusively of CKD G4–G5 non-dialysis patients, a population systematically excluded from pivotal phase III inclisiran trials.

Another noteworthy finding was the 27.4% reduction in albuminuria observed during follow-up. Albuminuria is a well-established marker of both renal and cardiovascular risk in CKD, and its reduction is generally associated with improved long-term outcomes (12). Nevertheless, these findings should be interpreted with considerable caution. Due to the retrospective nature of the study and the small sample size, causal inference cannot be established. In addition, important confounding variables, such as body weight changes during follow-up, were not systematically recorded. Furthermore, reductions in albuminuria may have been influenced by concomitant nephroprotective therapies, including renin–angiotensin system blockade, SGLT2 inhibitors, and finerenone. Importantly, however, all patients were already receiving maximally tolerated renin–angiotensin system blockade and SGLT2 inhibitors at study inclusion, with some additionally treated with finerenone, suggesting that the observed reduction in albuminuria occurred in the context of optimized background nephroprotective therapy. Therefore, the albuminuria reduction observed in this series should be considered hypothesis-generating only, and further prospective studies are needed to determine whether inclisiran may have renal effects beyond LDL-C lowering.

Interpretation of lipid parameters in advanced CKD also requires caution because of the so-called “lipid paradox” or “reverse epidemiology,” in which lower LDL-C levels may paradoxically associate with worse outcomes, often reflecting malnutrition, inflammation, or protein-energy wasting rather than true cardiovascular protection (13, 14). In our cohort, however, LDL-C reduction occurred in clinically stable patients without evidence of overt systemic illness progression, supporting the interpretation that the observed lipid lowering reflected a genuine pharmacological effect.

Finally, the twice-yearly dosing regimen of inclisiran may represent a practical advantage in advanced CKD patients, who frequently experience polypharmacy and medication adherence challenges. Simplified administration schedules and lower visit-to-visit variability in LDL-C levels could potentially contribute to improved long-term lipid control in this complex population (17, 19). In this context, inclisiran's unique pharmacological mechanism—a hepatocyte-specific siRNA that suppresses PCSK9 synthesis rather than circulating PCSK9 protein—confers a prolonged intracellular duration of action that sustains LDL-C lowering for over 6 months after a single dose, even though the drug itself clears from the systemic circulation within 48 h. As comprehensively reviewed by Di Giacomo-Barbagallo et al., this pharmacokinetic profile, combined with a consistent approximately 55% reduction in LDL-C, a robust safety record, and the ability to achieve reductions exceeding 80% when combined with other lipid-lowering therapies, positions inclisiran as a valuable addition to contemporary lipid-lowering strategies, particularly in patients in whom high therapeutic burden and adherence barriers limit the effectiveness of conventional approaches (20).

The main limitations of our study include its retrospective design, small sample size, and absence of a control group, which limit the generalizability of the findings and preclude causal inference. Additionally, although renal function and albuminuria were systematically collected, their evolution may have been influenced by concomitant therapies and biological variability. Nevertheless, to our knowledge, this study represents one of the first reports specifically evaluating inclisiran in a cohort composed entirely of patients with advanced CKD (eGFR < 30 ml/min/1.73 m^2^ not on dialysis), providing clinically relevant real-world safety data in a population largely excluded from pivotal clinical trials.

## 5 Conclusion

In this real-world case series of patients with advanced CKD (stages G4–G5 not on dialysis), inclisiran achieved a substantial and sustained reduction in LDL-C levels with an overall favorable safety profile. Kidney function remained globally stable during follow-up, and no major adverse renal events were observed, supporting the tolerability of inclisiran in patients with severe renal impairment.

Additionally, a reduction in albuminuria was observed despite optimized background nephroprotective therapy, including maximally tolerated renin–angiotensin system blockade and SGLT2 inhibitors. Although this finding should be interpreted cautiously due to the retrospective design and potential confounding factors, it raises the hypothesis of possible renal benefits beyond lipid lowering.

Given the limited representation of patients with eGFR < 30 ml/min/1.73 m^2^ in pivotal inclisiran clinical trials, our findings provide relevant preliminary real-world evidence supporting the safety of inclisiran, potentially representing a valid additional therapeutic option for reducing LDL cholesterol levels in patients at high and very high cardiovascular risk, such as those with advanced kidney disease. Larger prospective studies specifically including this population are needed to better define its long-term cardiovascular and renal effects.

## Data Availability

The datasets generated and analyzed during the current study are not publicly available due to patient privacy and ethical restrictions. De-identified data supporting the conclusions of this article are available from the corresponding author upon reasonable request and subject to approval by the relevant institutional authorities.
